# Sarcocystosis in Farm Animals in Brazil: A One-Health Approach

**DOI:** 10.3390/vetsci12090842

**Published:** 2025-09-01

**Authors:** Michel dos Santos Pinto, João Alfredo Biagi Camargo Neto, Carlos Wilson Gomes Lopes, Fernando Paiva, Luiz Daniel de Barros, Gustavo Felippelli, Fernando de Souza Rodrigues, Giovanni Widmer, Katia Denise Saraiva Bresciani

**Affiliations:** 1 Faculdade de Medicina Veterinária, Universidade Estadual Paulista (UNESP), Araçatuba 16050-680, São Paulo, Brazil; ms.pinto@unesp.br (M.d.S.P.); joao.alfredo@unesp.br (J.A.B.C.N.); gustavo.felippelli@unesp.br (G.F.); katia.bresciani@unesp.br (K.D.S.B.); 2 Instituto de Medicina Veterinária, Universidade Federal Rural do Rio de Janeiro (UFRRJ), Seropédica 23890-000, Rio de Janeiro, Brazil; lopescwg@ufrrj.br; 3 Instituto de Biociência, Universidade Federal do Mato Grosso do Sul (UFMS), Campo Grande 79070-900, Mato Grosso do Sul, Brazil; fernando.paiva@ufms.br; 4 Departamento de Medicina Veterinária, Universidade Federal de Lavras (UFLA), Lavras 37200-000, Minas Gerais, Brazil; 5 Departamento de Medicina Veterinária Preventiva, Universidade Estadual de Londrina (UEL), Londrina 86057-970, Paraná, Brazil; fernandosr@uel.br; 6 Cummings School of Veterinary Medicine, Tufts University, North Grafton, MA 01536, USA; giovanni.widmer@tufts.edu

**Keywords:** carnivores, epidemiology, horse, ruminants, zoonoses

## Abstract

Sarcocystosis is a parasitic infection caused by obligate intracellular coccidia, which infect humans, domestic animals, and wildlife. More than 200 *Sarcocystis* species have already been identified, but for many of these, the life cycle, pathogenesis, and clinical signs remain unclear. The infection is cosmopolitan, with high prevalence in cattle herds worldwide. Although the clinical disease in definitive hosts is considered rare, the high number of sporocysts released by them drives the incidence in production animals. Furthermore, sarcocystosis has some One Health relevance due to its zoonotic potential, especially concerning species infecting primates. Few studies have reported on the epidemiology of sarcocystosis in Brazil. However, a high prevalence of the disease was found in areas where investigations of *Sarcocystis* species were conducted, which highlights the potential for foodborne transmission to humans. Therefore, it is relevant to study this parasitic disease so that control and prophylaxis measures can be adopted. This study aims to review the current state of knowledge on *Sarcocystis* spp. in farm animals in Brazil.

## Simple Summary

Sarcocystosis is a parasitic disease caused by various *Sarcocystis* species affecting birds, reptiles, and mammals. Some species have the ability to infect humans by zoonotic transmission. In Brazil, studies on the occurrence of these parasites in production animals are scarce and were mostly concentrated in the southern region of the country. In these areas, the prevalence of *Sarcocystis* spp. is high, particularly in cattle and sheep, increasing the risk of transmission to humans. Although diagnostic methods are established, no specific and effective treatment options exist to control sarcocystosis, highlighting the importance of prevention in controlling this disease in production herds. Here, we review the literature on the etiopathogenesis, epidemiology, diagnosis, and control of sarcocystosis in production animals in Brazil.

## 1. Introduction

Species in the genus *Sarcocystis* (Lankester, 1882) were first described 150 years ago. These parasites are obligate intracellular coccidia belonging to the Apicomplexa phylum. Among pathogenic protozoa, Eimeriidae and Sarcocystidae are important families in terms of medical and veterinary interest, as they have the ability to parasitize a wide range of host species, including mammals, birds, fish, and reptiles [1]. In addition to a broad host range, sarcocystosis is distributed worldwide [2].

*Sarcocystis* spp. have a heteroxenic life cycle. The asexual stages are found in herbivores and omnivores, which serve as intermediate hosts, and the sexual stages in carnivores, the definitive host. Transmission relies on a prey–predator relationship between an intermediate host and a definitive host [3]. Concerning clinical manifestations, carnivores may present with self-limiting diarrhea, whereas herbivores may develop fever, anemia, vascular, reproductive, and neurological pathology [3,4,5]. The severity of the disease in the intermediate host depends on the number of sporocysts ingested. However, species that have dogs as their definitive host are comparatively more pathogenic than those that infect humans and felines [2].

Brazil has a high level of prevalence of sarcocystosis in herds, which increases the risk of transmission to humans of all ages [2,6]. Species of potential zoonotic concern include *Sarcocystis suihominis*, transmitted from swine to *Pan troglodytes*, *Macaca mulatta*, *Macaca irus* [7], and *Sarcocystis hominis* (syn. *Isospora hominis*), transmitted from cattle to rhesus monkeys (*Macaca mulata*) [8]. Data on *Sarcocystis* spp. in this country are still scarce [9]. To inform veterinarians and other health professionals, we report current information on sarcocystosis in farm animals.

## 2. Material and Methods

In this research, a narrative review of the literature was carried out through the analysis, interpretation, and synthesis of bibliographic information regarding sarcocystosis in production animals in Brazil. Bibliographic searches were conducted in PubMed, Scielo (Scientific Electronic Library Online), and the Periódico Capes database. The following descriptive terms were used as keywords: *Sarcocystis*, sarcocystosis, dog, cat, cattle, horse, swine, sheep, chickens, and Brazil. The inclusion criterion was that studies should report on *Sarcocystis* spp. in farm animals, in one or multiple host species, and only in this country. Studies related to farm animals, but focusing on other diseases, or reporting from other countries, were excluded. Therefore, information on articles published in scientific journals with reputable editorial policies was selected, interpreted, and compiled in this review. We used QGIS 3.28.9 software with freely accessible shapefiles from the Brazilian Institute of Geography and Statistics (IBGE) from 2023. The images were edited using the Canva graphic design platform (https://www.canva.com/).

## 3. Results

### 3.1. History

The first description of the *Sarcocystis* was made in 1843 by Miescher [10], who identified cysts in the striated muscles of house mice (*Mus musculus*), naming the parasite “Miescher’s tubules”. Similar structures were observed in pigs in 1865, but the name *Sarcocystis*, which derives from the Greek, *Sarkos*, meaning meat, and *Kystis*, cysts, was only proposed 34 years later. For a long time, due to the contamination of cultures and the appearance of hyphae and mycelia, these coccidia were classified in the Fungi kingdom. In 1967, 124 years after the first record, it became possible to investigate, using electron microscopy, the fusiform structures in the muscle cysts (bradyzoites) and observe that the organelles in these parasites were similar to those in the apicomplexan phylum, leading to its classification in the kingdom Protista [11]. Due to the original monotypy, the *Sarcocystis* type species is *S. miescheriana* [12].

### 3.2. Etiology

According to the taxonomic classification, *Sarcocystis* species belong to the kingdom Protista, phylum Apicomplexa, class Sporozoasida, subclass Coccidiasina, order Eucoccidiorida, suborder Eimeriorina, family Sarcocystidae, subfamily Sarcocystinae, genus *Sarcocystis* [13,14]. Currently, more than 200 species of *Sarcocystis* have been identified. Species differ in morphology, pathogenicity, and life cycle, although the latter two characteristics are only known for 26 species [2,3]. It has been demonstrated that humans can accidentally serve as intermediate hosts for several species of *Sarcocystis*, with the life cycle and definitive hosts being unknown in these cases [1]. The main species infecting farm animals are shown in Table 1.

### 3.3. Biological Cycle

**Figure 1:**
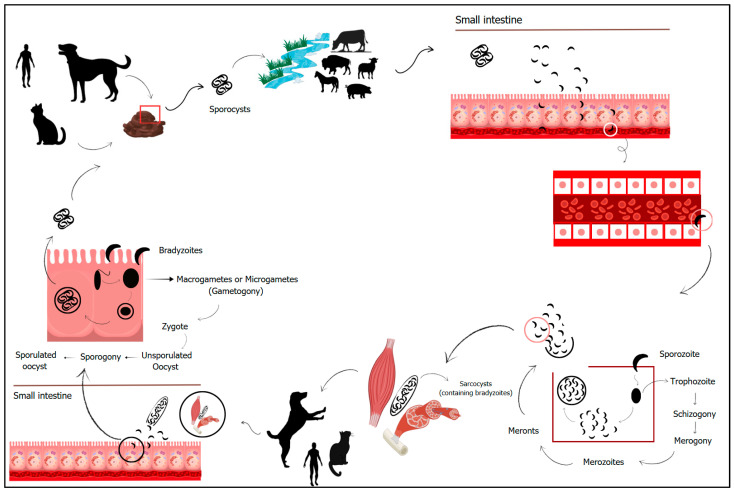
Biological cycle of Apicomplexa protozoa of genus *Sarcocystis*, including definitive hosts (carnivores/omnivores) and intermediate hosts (herbivores). Sporocysts are released in the feces of definitive hosts. Intermediate hosts become infected by ingesting sporocysts along with food and water. In these hosts, asexual reproduction occurs within the endothelial cells through the processes of schizogony and merogony. Merozoites are released into the bloodstream, migrate to the intermediate host’s musculature, and form sarcocysts filled with bradyzoites. Definitive hosts become infected by ingesting the intermediate host’s musculature containing the sarcocysts. Sexual reproduction of the agent occurs within the intestinal cells of definitive hosts, culminating in the formation of oocysts that contain sporocysts, which are released into the environment along with the feces.

Members of the genus *Sarcocystis* have a heteroxenic life cycle, with an asexual stage in intermediate hosts (herbivores or omnivores) and a sexual stage in definitive hosts (carnivores). Transmission from intermediate to definitive host depends on predation [3]. The infection occurs following ingestion of muscles from the intermediate host parasitized with sarcocysts containing bradyzoites. In the definitive host, after ingestion, bradyzoites penetrate the lamina propria of the intestinal mucosa and transform into macrogametes (female gametes) and microgametes (male gametes). The latter move to the periphery of the female gametes, culminating in fertilization and the formation of a wall around the zygote, which then forms the oocyst [3,4]. Oocysts excreted in the feces of the definitive host contain two sporocysts, each with four sporozoites [3]. Intermediate hosts become infected by ingesting *Sarcocystis* oocysts or sporocysts eliminated in the feces of the definitive host. In the digestive system of the intermediate host, the sporocysts rupture, releasing infectious sporozoites, which penetrate the intestinal mucosa and spread throughout the vascular system. The attachment to and penetration of the parasite into host cells occurs through the sporozoite’s apical complex, an organelle found in all coccidia [15]. Development takes place within the endothelial cells of the blood vessels [14]. Within the endothelial cells, sporozoites undergo a process of multinucleation (schizogony), transforming into meronts (merogony). Meronts give rise to merozoites, which can be released into the bloodstream through the rupture of the host cells. Merozoites then penetrate skeletal or cardiac striated muscle cells, forming sarcocysts full of bradyzoites (Figure 1). Three generations of merogony have been described. The heart, esophagus, diaphragm, and tongue are the most commonly affected organs, but encystation can occur in any muscular tissue [16].

### 3.4. Epidemiology

*Sarcocystis* is a cosmopolitan parasite with high prevalence in production herds. Factors such as a large number of definitive hosts, a high number of oocysts and sporocysts released by them, the ability of sporocysts to survive in the environment, and the capacity of intermediate hosts to harbor more than one species of *Sarcocystis* favor the dissemination [6]. In Brazil, few studies on *Sarcocystis* infection have been reported [6]; however, a high prevalence of the infection in cattle has been reported in the southern and northern regions of Brazil [6,17]. Table 2 and Table 3 include studies on the occurrence of *Sarcocystis* in animals in this country. In open-air markets in the northeast of the country, *Sarcocystis* cysts have been found in 100% of bovine hearts [18]. Sheep in the southern states of Santa Catarina and Rio Grande do Sul are also frequently infected [9]. Buffaloes also contribute to the transmission of sarcocystosis. Like other herbivores, these animals serve as an intermediate host. *Sarcocystis fusiformes*, *Sarcocystis buffalonis*, *Sarcocystis levinei*, and *Sarcocystis dubey* are the known parasites of buffaloes. In experimental studies, *Sarcocystis cruzi* and *Sarcocystis hominis* also demonstrated the ability to parasitize these animals [19].

**Figure 2:**
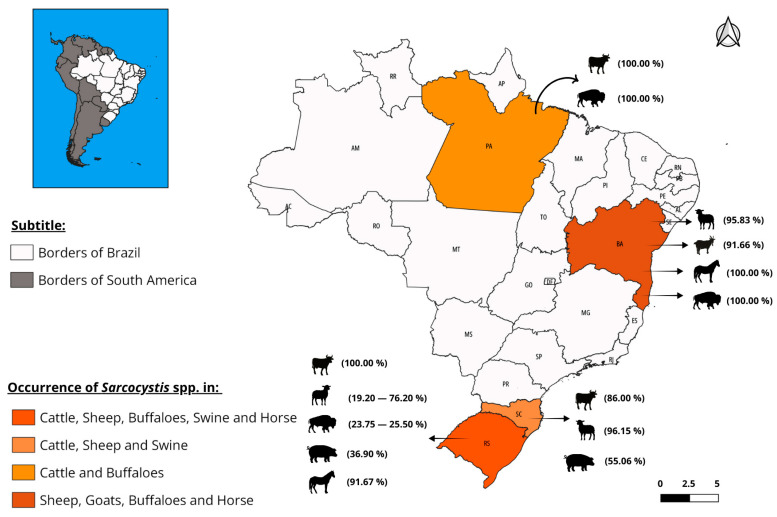
Spatial distribution of the occurrence of *Sarcocystis* spp. in cattle, sheep, buffaloes, pigs, and horses according to studies carried out in Brazil. The parasite’s occurrence is well-reported in the southern region of the country, particularly in the states of Rio Grande do Sul (RS) and Santa Catarina (SC). The Central-West (states of Goiás [GO], Mato Grosso [MT], and Mato Grosso do Sul [MS]) and Southeast (states of São Paulo [SP], Minas Gerais [MG], Espírito Santo [ES], and Rio de Janeiro [RJ]) regions are poorly studied. Map created with QGIS 3.28.9 software, using freely accessible shapefiles from the Brazilian Institute of Geography and Statistics (IBGE) from 2023.

In Brazil, reports on muscular sarcocystosis in horses are scarce. The first identification of *Sarcocystis* spp. in horses in the Americas was reported from the state of Bahia, where 100% of animals tested positive by parasitological examination. In this study, the species *Sarcocystis bertrami* was detected by molecular analysis [20]. In the state of Rio Grande do Sul, using a nested polymerase chain reaction (PCR) of the 18S ribosomal RNA gene, *Sarcocystis* spp. was detected in 91% of horses. Using Restriction Fragment Length Polymorphism (RFLP) and DNA sequencing, the species were identified as *Sarcocystis neurona* and *S. bertrami*, respectively [21]. Reports on sarcocystosis in pigs in Brazil are restricted to the southern region of the country (Figure 2), with only two epidemiological studies in the literature, showing a prevalence of 55% in Santa Catarina [22] and 37% in Rio Grande do Sul [23] (Table 2 and Figure 2).

To date, epidemiological aspects of *Sarcocystis* spp. in production chickens in Brazil have not been reported. However, in the Central-West region of the country, two backyard chickens were identified with necrotizing meningoencephalitis associated with an undescribed species of *Sarcocystis* [24]. However, *Sarcocystis*-associated encephalitis is a very rare form of sarcocystosis in chickens [24], and clinical disease in these animals is apparently atypical [25].

**Table 2:** Occurrence of *Sarcocystis* spp. in farm animals in different Brazilian states.

Year	Species	State	Nº Examined Animals	Diagnostic Method	Nº Positive Animals	Prevalence (%)	Authors
2015	Cattle	Pará	200	Peptide digestion and Scarification	200	100	Mangas et al., [6]
2018	Cattle	Rio Grande do Sul	314	Scarification	314	100	Ferreira et al., [26]
2019	Cattle	Santa Catarina	146	Histopathology	122	86.00	Quadros et al., [2]
2016	Sheep	Rio Grande do Sul	80	Scarification	61	76.20	Portella et al., [27]
2016	Sheep	Bahia	120	Tissue squash and Tissue grinding	115	95.83	Bittencourt et al., [28]
2018	Sheep	Rio Grande do Sul	161	Macroscopic and Histopathology	31	19.20	Panziera et al., [29]
2019	Sheep	Santa Catarina	130	Tissue grinding	125	96.15	Minuzzi et al., [9]
2016	Goats	Bahia	120	Tissue squash and Tissue grinding	110	91.66	Bittencourt et al., [28]
1996	Buffaloes	Bahia	43	Scarification	33	76.74	Rebouças et al., [30]
2016	Buffaloes	Rio Grande do Sul	220	IFAT	56	25.50%	Portella et al., [31]
2016	Buffaloes	Pará	100	Peptide digestion	100	100	Rabello, [32]
2021	Buffaloes	Rio Grande do Sul	80	Tissue grinding	19	23.75	Portella et al., [33]
2019	Swine	Santa Catarina	296	Histopathology	163	55.06	Morés et al., [22]
2022	Swine	Rio Grande do Sul	84	IFAT	31	36.90	Espindola et al., [23]
2022	Horse	Bahia	51	Tissue grinding	51	100	Marques et al., [20]
2024	Horse	Rio Grande do Sul	24	PCR	22	91.67	Rosa et al., [21]

Indirect Fluorescent Antibody Test (IFAT); Polymerase Chain Reaction (PCR).

**Figure 3:**
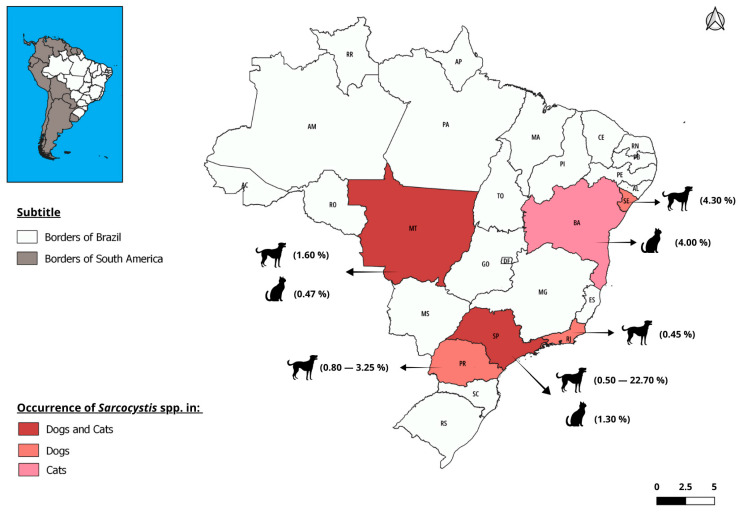
Spatial distribution of the occurrence of *Sarcocystis* spp. in dogs and cats according to studies carried out in Brazil. The occurrence of *Sarcocystis* spp. was reported in six states, including São Paulo (SP), Paraná (PR), Rio de Janeiro (RJ), Mato Grosso (MT), Bahia (BA), and Sergipe (SE). Map created with QGIS 3.28.9 software, using freely accessible shapefiles from the Brazilian Institute of Geography and Statistics (IBGE) from 2023.

Regarding the definitive host, dogs play an important role in the transmission of the infection, as they are in direct contact with production herds. This is not the case for felines since they live almost exclusively indoors [6]. The prevalence of *Sarcocystis* in dogs and cats in Brazil appears to be low (Figure 3). However, few coproparasitological studies have been conducted to diagnose this parasite in these host species (Table 3).

**Table 3:** Occurrence of *Sarcocystis* spp. in dogs and cats in different Brazilian regions.

Year	Species	State	Nº Examined Animals	Diagnostic Method	Nº Positive Animals	Prevalence (%)	Authors
2002	Dogs	São Paulo	271	FSSCS and CFSZSS	6	2.20	Oliveira et al., [34]
2014	Dogs	Sergipe	93	FSSCS and SS	4	4.30	Lima et al., [35]
2015	Dogs	Rio de Janeiro	221	CFSS and Direct examination	1	0.45	Leal et al., [36]
2015	Dogs	Paraná	123	FSSCS and SS	4	3.25	Ribeiro et al., [37]
2016	Dogs	São Paulo	3099	CSWE, CFSS, and FSSCS	16	0.50	Ferreira et al., [38]
2016	Dogs	São Paulo	1000	FSSCS, CFSS, and CSFES	37	3.70	Lallo et al., [39]
2017	Dogs	Mato Grosso	120	CFSZSS and SS	2	1.60	Lima & Malheiros, [40]
2017	Dogs	Paraná	120	FSSCS, SS, and CFSS	1	0.80	Snak, [41]
2018	Dogs	São Paulo	22	CFSS	5	22.70	Sevá et al., [42]
2016	Cats	São Paulo	502	CSWE, CFSS and FSSCS	7	1.30	Ferreira et al., [38]
2016	Cats	Mato Grosso	210	CSWE and SS	1	0.47	Lins, [43]
2014	Cats	Bahia	272	IFAT	11	4.00	Meneses et al., [44]

Centrifugal Sedimentation in Water-Ether (CSWE). Centrifugal Flotation in Sucrose Solution (CFSS). Flotation in Saturated Sodium Chloride Solution (FSSCS). Centrifugal Flotation in Saturated Zinc Sulfate Solution (CFSZSS). Spontaneous Sedimentation (SS). Centrifugal Sedimentation in Formalin-Ether Solution (CSFES). Indirect Fluorescent Antibody Test (IFAT).

### 3.5. Pathogenesis and Clinical Signs

*Sarcocystis* species are considered more pathogenic to intermediate hosts than to definitive hosts, but species that infect dogs are reported to be more virulent than species that infect felines [4]. After ingesting the sporocysts, the sporozoites are released into the intestinal tract of intermediate hosts, where they develop asexually within the endothelial cells. Intracellular development compromises local blood flow due to the increase in infected cells and lesions in the cell wall caused by the release of merozoites, as well as an extensive reaction similar to disseminated intravascular coagulation, characterized by platelet aggregation and the formation of thrombi at injured sites [45]. The merozoites resulting from the infection of the intimate tunica of the vessels penetrate muscle cells, where they encyst. This stage in the life cycle is characterized by elevated serum enzymes, such as aspartate aminotransferase, creatine kinase isoenzymes, and lactate dehydrogenase [17].

The pathogenesis in definitive hosts is poorly understood, with few published reports. In a histopathological study of the small intestine and mesenteric lymph nodes of dogs infected with *S. cruzi* in Barra Mansa in the state of Rio de Janeiro, a slight edema and plasma cell infiltrate were observed in the mucous membranes of the duodenum, jejunum, and ileum, consistent with a minimal inflammatory reaction. Lymph nodes showed cellular rarefaction in the medullary region, and some primary follicles were present in the cortical region, close to the afferent lymphatic vessels that drain all the lymph in the region. The presence of sporulated oocysts was observed. This pathology is consistent with the involvement of lymphatic vessels in the dissemination of sporocysts and sporozoites through mesenteric lymph nodes [46]. The clinical signs observed in ruminants are discreet and, when present, include fever, anorexia, anemia, emaciation, weight loss, alopecia at the base of the tail, neurological signs such as weakness of the hind limbs, ataxia, paresis, and myopathy, generally three to four weeks after ingestion of the sporocysts. As the sarcocysts mature, the symptoms become less evident. Acute cases are characterized by reproductive disorders, such as abortion and even mortality, depending on the level of infection and the immune status of the host [3,4,5]. The severity of the disease depends on the number of sporocysts ingested. Dogs and cats generally do not present marked clinical manifestations of intestinal infection, the only sign being enteritis [47].

### 3.6. Zoonotic Aspects

Human sarcocystosis is caused by two species, *S. hominis* and *S. suihominis*. Humans can serve as the definitive host of these species and as accidental intermediate hosts for species that are still being studied and identified. In experimental studies, it has been reported that primates such as the chimpanzee (*Pan troglodites*) and rhesus monkey (*Macaca mulata*) can also act as definitive hosts of these species, both hosts becoming infected through ingestion of raw or “undercooked” meat of cattle and pigs infected with sarcocysts [15,16].

The pre-patent period of this disease is around 10 days, which generally causes minimal damage to the intestinal mucosa. However, the infection with *S. suihominis* tends to be more serious. Clinical signs, when present, are similar to those of transient enteritis, including nausea, abdominal pain, and diarrhea [1]. There are reports in the literature that intestinal infections by *S. hominis* and *S. suihominis* often lead to immunity capable of reducing the severity of the disease in these hosts without preventing new infections [48]. The main species causing muscular sarcocystosis in humans is *Sarcocystis nesbitti* [49], and the primary clinical signs include persistent myalgia, muscle weakness, dermatomyositis, subcutaneous nodules, fever, transient lymphadenopathy with eosinophilia, and increased levels of creatine kinase. In such cases, the infection results from the ingestion of water and food contaminated with oocysts/sporocysts [50]. The consumption of raw or undercooked meat contaminated with *Sarcocystis* cysts other than *S. hominis* or *S. suihominis* is also of epidemiological importance since the cysts contain a neurotoxin called sarcocystin, which alters cell membranes and leads to excessive water uptake and the increased release of inflammatory mediators. These processes can result in hyperthermia and gastroenteric disorders. It is worth mentioning that in studies with rabbits, sarcocystin inoculation was lethal [48].

The high prevalence of *S. bertrami* in horses in the state of Bahia [20] and in Rio Grande do Sul [21] highlights the risk to public health posed by this species, as *S. bertrami* cysts have been identified as causing 27 outbreaks of food poisoning in Japan [51,52]. In Brazil, the consumption of horse meat is uncommon; however, the country is considered the sixth-largest slaughterer of horses [53]. Horse meat is exported to several countries in Europe and Asia [54], where it is also consumed raw, which can increase the risk of food poisoning by *S. bertrami*.

### 3.7. Diagnosis

Diagnostic methods for sarcocystosis are different and depend on the host. Oocysts and sporocysts shed by the definitive host are diagnosed with coprological examination. Microscopy is used to detect sporocysts or oocysts following flotation on a saturated solution of sucrose or zinc sulfate [4,55]. The diagnosis of muscular sarcocystosis in production animals is described in the manual of the World Organization for Animal Health (WOAH). In Brazil, post-mortem diagnosis is carried out during official health inspection, in accordance with regulation number 168/2020 of Regulation of Industrial and Sanitary Inspection of Products of Animal Origin, by visual inspection and incision of the internal and external masseter muscles of animals older than six months, and by inspecting longitudinal sections of the heart [56]. The IFAT and Enzyme-Linked Immunosorbent Assay (ELISA) tests are serological tests used to detect antibodies against *Sarcocystis* spp. Since they are not species-specific, and there is a likelihood of cross-reactivity between different species, these tests are used as a screening tool. Furthermore, due to the lack of specificity of serological tests, molecular diagnostic methods have been increasingly used to diagnose individual *Sarcocystis* species. Given their similar morphologies, microscopy and serology cannot differentiate between species [3,10].

The primary molecular technique for diagnosing and identifying species of *Sarcocystis* is PCR, including nested PCR and the RFLP technique. The 18S rRNA gene is the main genetic marker used in these applications [17,21,57,58,59,60,61]. It is worth noting that microscopic examination of sarcocysts, PCR, and serology present good sensitivity and agreement in the diagnosis of *Sarcocystis* spp., which can facilitate diagnosis in live animals and help in herd monitoring [17].

### 3.8. Treatment

Intestinal sarcocystosis is a self-limiting infection, and to date, there is no effective treatment for humans or veterinary applications. Studies evaluating potential drugs, such as co-trimoxazole [62] and furazolidone [63], have shown inconclusive results. These findings also apply to therapies directed at muscular sarcocystosis. The use of corticosteroids as immunosuppressants has been shown to improve symptoms of myositis and reduce the inflammatory reaction in cases of vasculitis. However, immunosuppression can favor and prolong the parasitic infection [8].

### 3.9. Prevention and Control

No vaccines against sarcocystosis are available. The most effective prophylaxis is interrupting the life cycle by avoiding contact between the definitive host and facilities where production animals are raised. Not feeding raw meat and offal to dogs and cats, and burying carcasses of animals that die in the field are effective preventive measures [4]. Preventing infection in the field becomes very difficult, as it is intrinsically linked to the transmission of the merogonic stages by hematophagous insects between an intermediate host and another of the same species.

Freezing meat at −4 °C or −20 °C for 48 or 24 h, respectively, or cooking meat at 60 °C, 70 °C or 100 °C for 20, 15 or 5 min, respectively, prevents foodborne transmission, as does water treatment and general sanitary and hygienic methods [14]. Health education and public awareness about the risk of fecal contamination of soil caused by fertilizers derived from human waste, as well as government inspection of animal products, are also preventive measures [2].

## 4. Conclusions

*Sarcocystis* is a parasite of potential One Health importance. There are few studies on the epidemiology, treatment, and prevention of the disease in Brazil. A high prevalence of the disease has been described in cattle herds in areas where the occurrence of this agent has been investigated, including in sheep, which increases the risk of human infection and food poisoning. Implementation of prophylactic measures depends on a better understanding of these parasites.

## Figures and Tables

**Table 1:** Species of the genus *Sarcocystis* identified in humans and domestic animals.

Definitive Hosts	Intermediate Hosts						
	Cattle	Buffaloes	Sheep	Goat	Horse	Swines	Chickens
Dogs	*S. cruzi* *	*S. levini* *	*S. tenella* * S. arienticanis *	*S. capracanis* * S. hircicanis	*S. bertrami* *	*S. miescheriana* *	*S. wenzeli*
Cats	*S. hirsuta* *S. bovifelis* *S. rommeli*	*S. fusiformis* *S. buffalonis*	*S.gigantea* * S. medusiformis	*S. moulei*		*S. porcifelis*	*S. wenzeli*
Humans	*S. hominis* * S. heydorni					*S. suihominis* *	
Unknown	*S. bovini*	*S. dubeyi*					

* Species identified in Brazil. S.—*Sarcocystis*. Source: Adapted from [2,10,13].
